# Revisiting the Benefits of Exercise for Alzheimer's Disease through the Lens of Ferroptosis: A New Perspective

**DOI:** 10.14336/AD.2024.1560

**Published:** 2025-12-21

**Authors:** Zikang Hao, Xinmeng Guo, Jiawen Wu, Guang Yang

**Affiliations:** ^1^Department of Sport and Health, School of Physical Education, Shandong University, Jinan, China.; ^2^Kunshan Hospital Affiliated to Nanjing University of Chinese Medicine, Jiangsu, China.

**Keywords:** ferroptosis, exercise, Alzheimer's disease, oxidative stress, nrf2

## Abstract

Ferroptosis, an iron-dependent form of programmed cell death driven by oxidative stress, plays a crucial role in the progression of Alzheimer's disease (AD). Aging diminishes antioxidant systems that maintain iron homeostasis, particularly affecting the glutathione peroxidase (GPX) system, leading to increased ferroptosis and exacerbated neurodegeneration and neuroinflammation in AD. Nuclear factor erythroid 2-related factor 2 (Nrf2) is a key transcription factor regulating genes involved in antioxidant defense and ferroptosis. In this review, we examine the interconnected roles of Nrf2 signaling, iron metabolism, and ferroptosis in AD, and discuss how regular physical exercise—known to enhance antioxidant capacity—might influence these processes. Despite evidence linking exercise to improved cognitive function in AD and its role in modulating oxidative stress, there is a paucity of research specifically addressing how exercise affects ferroptosis in the AD brain. To address this gap, we utilized bioinformatics techniques to identify potential pathways and mechanisms by which exercise may mitigate ferroptosis in AD through Nrf2 signaling. Analyzing gene expression profiles from the GEO database, we identified differentially expressed ferroptosis-related genes in the hippocampus following exercise intervention. Hub genes like SLC2A1, TXN, MEF2C, and KRAS were significantly upregulated, suggesting that exercise may activate a network enhancing antioxidant defenses and regulating iron metabolism via Nrf2. Our findings propose a novel mechanism whereby exercise alleviates abnormal ferroptosis in the AD brain through modulation of Nrf2 signaling. This study highlights the need for further research to validate these findings and explore exercise as a therapeutic strategy for AD by targeting ferroptosis.

## 1. Iron metabolism and Ferroptosis

## 1.1 Iron Metabolism

Iron is the second most abundant metal in the Earth's crust, after aluminum, and is the most abundant transition metal in biological systems. Iron plays a crucial role in various essential biological processes through its different oxidation states, including oxygen transport, DNA synthesis and repair, respiratory activities, myelin synthesis, and cellular metabolism [1]. Iron homeostasis is maintained by several mechanisms, such as iron regulatory proteins (IRPs) that regulate both systemic and cellular functions. Disruption of iron homeostasis can lead to excessive intracellular iron accumulation, causing damage to proteins, lipids, and DNA through the generation of free radicals and oxidative stress [2, 3]. More importantly, iron is essential for the synthesis of myelin and neurotransmitters in the central nervous system (CNS), which are critical for maintaining CNS function. However, iron also acts as a double-edged sword; excessive iron concentrations, free iron ions, and disruption of iron metabolism contribute to the progression of CNS-related diseases [4, 5].

## 1.2 Ferroptosis and Its Mechanisms

**Figure 1.**
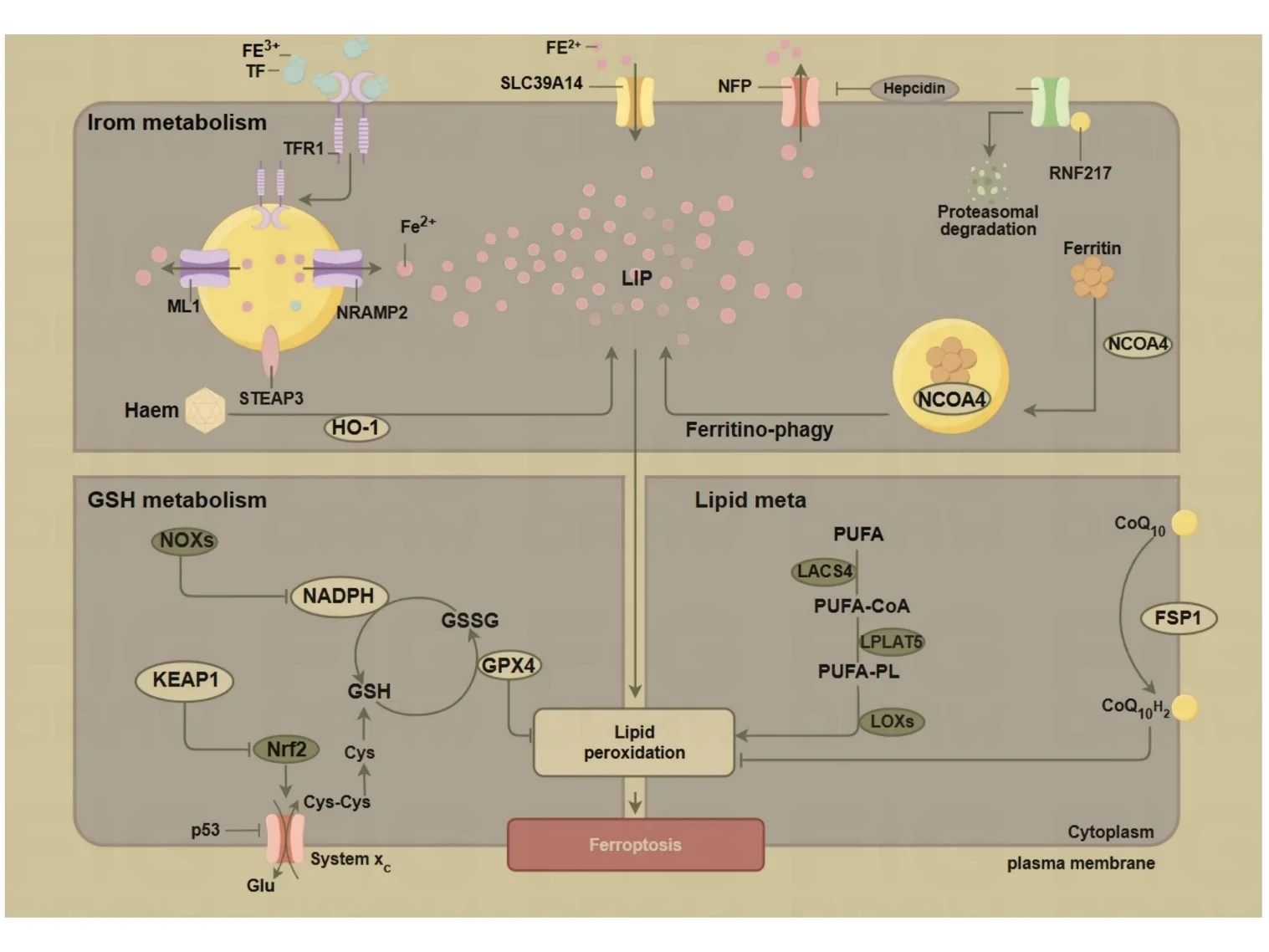
**Mechanism of Ferroptosis**. Iron Metabolism: Extracellular ferric iron (Fe³^+^) is transported into cells via TFR1 and reduced to ferrous iron (Fe²^+^) by STEAP3. The reduced Fe²^+^ is released into the cytosolic labile iron pool (LIP) through NRAMP2. Ferritin stores excess Fe²^+^, but under the regulation of NCOA4, ferritinophagy releases stored iron back into the LIP. This increase in free iron accelerates its accumulation and ultimately triggers ferroptosis. GSH Metabolism: GSH is a key antioxidant that inhibits ferroptosis by reducing lipid peroxides via GPX4. GSH synthesis depends on cysteine (Cys) supplied by the System xc^-^ antiporter. Additionally, Nrf2 activates genes involved in GSH synthesis, while NOXs promote the production of ROS, depleting the cell's antioxidant defenses and exacerbating oxidative stress. Lipid Peroxidation: Polyunsaturated fatty acids (PUFAs) are converted into PUFA-phospholipids through the actions of LACS4 and LPLAT. These PUFA-phospholipids are then peroxidized by LOXs, leading to lipid membrane damage and the onset of ferroptosis. In contrast, the FSP1/CoQ10 pathway counteracts lipid peroxidation by generating reduced Coenzyme Q10 (CoQ10H_2_), thereby inhibiting ferroptosis.

Ferroptosis, identified in 2012, is a unique form of programmed cell death distinct from apoptosis, necrosis, and other cell death types. It is primarily driven by iron-dependent lipid peroxidation, which leads to the accumulation of reactive oxygen species (ROS) through Fenton reactions, resulting in oxidative stress and cell death [6]. As shown in Figure 1. For the time being, the three pathways that are clearer regarding the mechanism of ferroptosis are: Lipid Peroxidation, Amino Acid Metabolism Disruption and Iron Accumulation.

Iron Accumulation: Iron metabolism is intricately linked to ferroptosis. Dietary iron is absorbed by enterocytes and transported into the bloodstream via ferroportin 1 (FPN1) on the basolateral membrane. In the blood, ceruloplasmin oxidizes ferrous iron (Fe²^+^) to ferric iron (Fe³^+^), which then binds to transferrin for delivery to various tissues. Transferrin receptors 1 and 2 (TFR1/TFR2) on cell membranes mediate the uptake of iron into cells [7]. Excessive intracellular iron enhances the Fenton reaction, producing hydroxyl radicals (-OH) and increasing the labile iron pool (LIP) and ROS levels. These ROS interact with polyunsaturated fatty acids (PUFAs) in lipid membranes, initiating lipid peroxidation and leading to ferroptosis [8, 9].

Lipid Peroxidation: Central to ferroptosis is the peroxidation of lipids, particularly targeting polyunsaturated phosphatidylethanolamines (PEs) such as arachidonic acid (AA) and adrenic acid (AdA). The enzyme long-chain acyl-CoA synthetase 4 (ACSL4) facilitates the esterification of AA or AdA with coenzyme A (CoA), forming their respective CoA derivatives. These derivatives are then incorporated into PE by lysophosphatidylcholine acyltransferase 3 (LPCAT3) to produce AA-PE and AdA-PE [10]. Subsequently, 15-lipoxygenase (15-LOX) oxidizes these PEs, generating lipid hydroperoxides that propagate ferroptosis [11].

Amino Acid Metabolism Disruption: Amino acid metabolism plays a critical role in ferroptosis regulation. Glutathione peroxidase 4 (GPX4) is a key enzyme that mitigates lipid peroxidation by converting lipid hydroperoxides (L-OOH) into non-toxic lipids [12]. GPX4 activity depends on glutathione (GSH), which is synthesized from glutamate, cysteine, and glycine. The cystine/glutamate antiporter system Xc- (SLC7A11) imports cystine, a precursor for GSH synthesis [13, 14]. Inhibition of system Xc- depletes cysteine, reduces GSH levels, and compromises GPX4 function, thereby enhancing oxidative stress and promoting ferroptosis. Additionally, the tumor suppressor protein p53 can induce ferroptosis by downregulating SLC7A11, further disrupting GSH synthesis and GPX4 activity [15].

## 2. Ferroptosis in AD

## 2.1 Aging and Ferroptosis

Aging is a significant risk factor for AD [16]. As individuals age, the brain's ability to regulate iron metabolism and the functionality of the GPX antioxidant system declines. This reduction in antioxidant defenses disrupts iron homeostasis, leading to iron accumulation that promotes ferroptosis and accelerates AD progression [17, 18].

GPX4 is highly expressed in the brain and plays a critical role in AD. Mutations in the GPX4 gene increase the risk of developing AD, with the GPX4 rs713041 variant linked to a higher likelihood of the disease [19]. In mouse models, loss of GPX4 in forebrain neurons leads to age-related neurodegeneration and significant neuronal loss, highlighting GPX4's connection to AD [20]. In the hippocampus of AD patients, there is notable iron accumulation, lipid peroxidation, and reduced levels of both GSH and GPX4 [21]. Studies using GPX4 knockout mice demonstrate that cognitive deficits and neurodegeneration are associated with increased lipid peroxidation, activation of extracellular-regulated kinases (ERK1/2), and pronounced neuroinflammation, without caspase-3 activation [22]. These observations suggest that ferroptosis, rather than apoptosis, contributes to AD pathology. Therefore, ferroptosis induced by impaired GPX4 may be a key mechanism in AD neurodegeneration.

## 2.2 Aβ Deposition and Ferroptosis in AD

**Figure 2.**
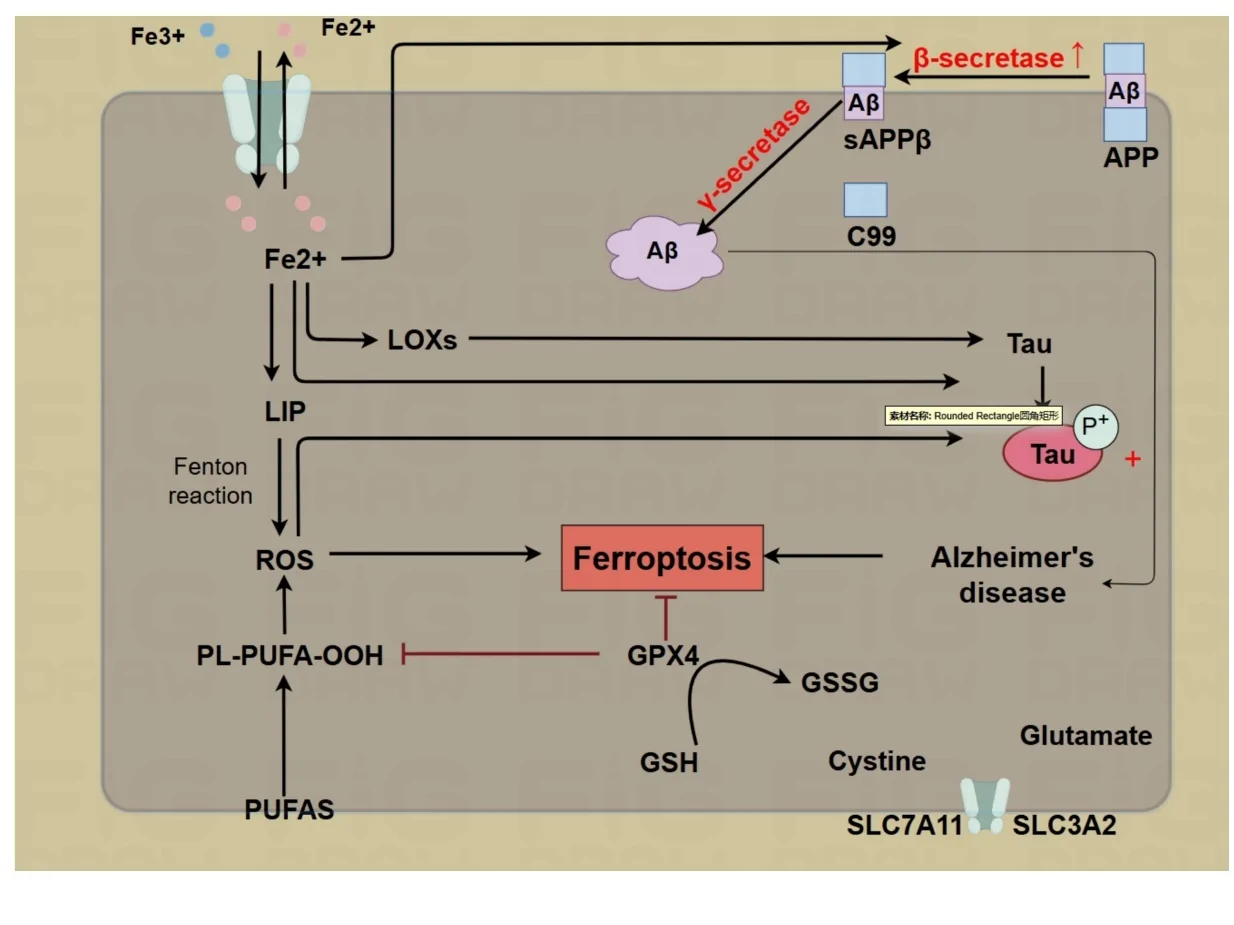
**The mechanism of ferroptosis in AD brain**. The interplay between ferroptosis mechanisms and the pathological feature of AD is evident. In summary, excessive intracellular iron accumulation enhances the activity of β-secretase and γ-secretase, promoting Aβ deposition. Additionally, the ROS generated by iron overload or lipid peroxides exacerbate the abnormal phosphorylation of tau protein, further accelerating AD progression. Moreover, neuronal damage caused by ferroptosis disrupts cognitive function, posing a significant challenge to its maintenance.

Research into AD pathogenesis has demonstrated a bidirectional relationship between excessive iron deposition and the accumulation of amyloid-beta (Aβ) plaques. As shown in Figure 2. Mutations in the APP primarily drive Aβ deposition, while iron acts as a key regulator of brain iron levels in this context [23]. Elevated iron not only increases APP expression but also enhances the activity of γ-secretase, an enzyme that promotes Aβ production [24]. Importantly, iron accumulation does not directly influence the expression of β-site APP cleaving enzyme 1 (BACE-1), which is responsible for the initial cleavage of APP. Instead, it creates a positive feedback loop where the initial formation of Aβ leads to increased BACE-1 expression in surrounding neuronal areas, further driving Aβ production [25, 26].

High levels of BACE-1 in AD patients result in reduced activity of superoxide dismutase 1 (SOD1), thereby increasing oxidative damage. Additionally, the buildup of intracellular iron enhances APP translation, leading to more Aβ deposition [27]. This regulation is mediated by the iron response element (IRE) located in the 5' untranslated region (5'UTR) of APP mRNA [28]. Post-translational modifications, such as phosphorylation, hinder APP translation from reaching the cell surface, which decreases the expression of FPN1. Reduced FPN1 impairs iron export, resulting in further intracellular iron accumulation [29]. Thus, the interaction between iron and APP is crucial in AD development.

Iron regulatory proteins (IRP1 and IRP2) play a vital role in maintaining iron homeostasis by interacting with IREs in the 5'UTR of APP transcripts. This interaction blocks IRP1/2 binding to IREs, thereby inhibiting APP translation and leading to increased iron levels [30]. Notably, mutations in APP can alter the localization of FPN1, affecting neuronal iron content. Furthermore, Aβ proteins bind significant amounts of metals, including iron, zinc, and copper, which can induce oxidative stress [17, 18]. The interaction between Aβ and these redox-active metals rapidly triggers the formation of AD-related proteins and toxic oligomers, resulting in the generation of reactive oxygen species (ROS). Among these metals, iron is the most abundant in the brain, and its redox activity is a primary contributor to the oxidative potential of Aβ.

## 2.3 Abnormal Phosphorylation of Tau Protein and Ferroptosis in AD

Under normal physiological conditions, tau protein is essential for transporting APP to the cell membrane, stabilizing FPN1, and facilitating iron efflux [31]. However, excessive post-translational modifications of tau—such as phosphorylation, acetylation, glycosylation, and ubiquitination—along with APP secretase cleavage, can lead to tau aggregation. This aggregation hinders APP from properly reaching the cell surface, resulting in decreased FPN1 levels [32]. Consequently, iron accumulates and aggregates in neurons, contributing to the formation of neurofibrillary tangles (NFTs).

Excessive neuronal iron further exacerbates tau phosphorylation. ROS generated by iron deposition can promote tau oligomerization by reacting with cysteine thiol groups [33]. Additionally, high iron levels may enhance tau hyperphosphorylation and NFT formation through signaling pathways involving cyclin-dependent kinase 5 (CDK5)/P25 complexes, glycogen synthase kinase-3β (GSK-3β), and protein phosphatase 2A (PP2A) [34]. Oxidative stress indirectly influences tau protein regulation as well.

Importantly, iron dysregulation triggers the Fenton/Haber-Weiss reaction, increasing oxidative free radicals and oxidative stress, which in turn stimulates tau hyperphosphorylation [35]. The formation of NFTs is closely linked to the redox state of iron. The transition from Fe³^+^ to Fe²^+^ iron can reverse tau aggregation. Iron forms coordination complexes with phosphorylated amino acid residues, promoting tau oligomer formation. Furthermore, Fe³^+^ enhances the pathological functionality of highly phosphorylated tau oligomers by aiding their binding to membrane lipids, thereby worsening disease progression [36, 37].

A recent study suggested that iron, through its binding motifs in tau and by disrupting insulin signaling, may lead to tau hyperphosphorylation and aggregation [38]. Proper expression of FPN1 is crucial for regulating intracellular iron efflux. However, excessive modification of tau decreases FPN1 stability and expression, causing iron accumulation and ROS generation. This creates a negative feedback loop that promotes further tau oligomerization [31]. Additionally, iron accumulation stimulates tau hyperphosphorylation mediated by activators such as GSK-3β and PP2A. The oxidative free radicals produced via the Fenton reaction and the resultant oxidative stress further exacerbate tau hyperphosphorylation.

In summary, disruptions in iron metabolism and ferroptosis interact with tau pathology in a negative feedback loop, collectively advancing the progression of Alzheimer's Disease.

## 2.4 Neuro Damage and Ferroptosis in AD

In the context of AD, neuronal injury is a central feature that contributes to cognitive decline and neurodegeneration. Recent studies have implicated ferroptosis, an iron-dependent form of regulated cell death, as a key mechanism underlying neuronal damage in AD brains [39]. Unlike other cell death pathways such as apoptosis or necrosis, ferroptosis is specifically characterized by iron accumulation, lipid peroxidation, and the depletion of antioxidant defenses, which collectively exacerbate neuronal injury.

Experimental models of AD have provided evidence supporting the involvement of ferroptosis in neuronal injury. In AD-affected brain regions, there is a significant increase in iron levels and lipid peroxidation markers, which correlate with the extent of neurodegeneration and cognitive deficits. Studies on AD mouse models have shown that inhibiting ferroptosis pathways can mitigate neuronal loss and improve cognitive function, underscoring the pathogenic role of ferroptosis in AD [40, 41]. Additionally, the expression of ferroptosis-related genes, such as those involved in iron metabolism and antioxidant defenses, is dysregulated in AD brains, further linking ferroptosis to the disease process [42].

Targeting ferroptosis presents a promising therapeutic strategy for protecting neurons in AD. Iron chelators, which reduce iron availability, have been shown to decrease lipid peroxidation and neuronal death in AD models [43]. Antioxidant therapies that restore GSH levels or activate GPX4 can also inhibit ferroptosis, thereby preserving neuronal integrity and function [44, 45]. Moreover, identifying and modulating specific regulators of ferroptosis may lead to the development of targeted interventions that prevent or slow the progression of neuronal injury in AD.

## 3. The Role of Resistance to Ferroptosis Represented by Nrf2 in AD

## 3.1 Crosstalk Between Nrf2 and Ferroptosis

Nrf2 is a pivotal transcription factor that regulates both iron metabolism and ferroptosis. Its downstream target genes include numerous proteins associated with ferroptosis, such as NADPH, heme oxygenase-1 (HO-1), SLC7A11/xCT, and various phase II detoxification enzymes like glutathione S-transferase (GST), UDP-glucuronosyltransferase, GPX4, glutathione reductase, and subunits of glutamate-cysteine ligase [46, 47]. Extensive studies have confirmed that Nrf2 plays a crucial role in managing iron levels and controlling ferroptotic processes, highlighting its importance in AD pathology.

Protein interaction network analyses have shown that Nrf2 directly regulates ferroptosis through GPX4-related enzymes, including glucose-6-phosphate dehydrogenase (G6PD), glutathione reductase (GSR), GPX4, glutamate-cysteine ligase modifier subunit (GCLM), SLC7A11, glutamate-cysteine ligase catalytic subunit (GCLC), and thioredoxin reductase 1 (TXNRD1). Additionally, Nrf2 influences ferroptosis via the peroxisome proliferator-activated receptor γ (PPARγ) pathway [56]. Beyond these direct interactions, Nrf2 indirectly regulates intracellular iron levels through the Nrf2-heme oxygenase 1 (HMOX1)-iron regulatory protein axis, which includes biliverdin reductase A/B (BLVRA/B), ferritin heavy chain 1 (FTH1), transferrin receptor 1 (TFRC), ferrochelatase (FECH), and ferroportin 1 (FPN1/SLC40A11). These pathways collectively modulate ferroptosis by maintaining iron balance within cells. Based on substantial evidence, it is reasonable to conclude that Nrf2 alleviates ferroptosis and maintains iron homeostasis, making it a critical factor in the progression of Alzheimer's Disease [48].

## 3.2 Targeting Nrf2 to Regulate Ferroptosis in AD Brain

Previous studies have identified oxidative stress in the CNS as a potent inducer and promoter of AD. Interestingly, a reduction in Nrf2 expression has been observed in AD patients, particularly within neuronal nuclei [49]. This decrease may result from impaired nuclear translocation of Nrf2 or reduced binding of Nrf2 to antioxidant response elements (AREs). These findings have been consistently observed in both human and animal models, establishing a causal relationship between diminished Nrf2 expression and increased BACE1 levels, enhanced Aβ deposition, and heightened neuroinflammation. The overactivation of GSK-3β is believed to contribute to this phenomenon [50]. Large-scale evidence-based studies have revealed that the expression of 31 Nrf2-dependent genes is downregulated in AD, partially explaining the oxidative stress observed in AD brains [51]. Additionally, investigations into Down syndrome patients, who exhibit AD-like dementia symptoms, have shown suppressed Nrf2 expression. This suppression is associated with elevated levels of BACH1, a key inhibitor of Nrf2 [52].

The expression levels of Nrf2 are closely linked to various pathogenic factors that drive AD progression. For instance, as previously mentioned, in physiological conditions, there exists a dynamic balance between NF-κB and Nrf2 to ensure the resolution of inflammation. Specifically, ROS-induced activation of NF-κB is counteracted by Nrf2, which prevents the degradation of IκB-α and increases the expression of HO-1, thereby inhibiting oxidative stress-induced neuroinflammation [53]. In AD brains, microglia and astrocytes, which are central to neuroinflammation, undergo proliferation—a process known as gliosis [54]. Studies have shown that APP/PS1 mice with Nrf2 deficiency exhibit more severe neuroinflammatory responses. Furthermore, in mice with Nrf2 deficiency and brain-targeted injection of human Tau adenovirus, there is a significant increase in the proliferation of Iba-1-positive microglia and GFAP-positive astrocytes, along with elevated secretion of pro-inflammatory cytokines compared to normal mice [55]. Conversely, activation of Nrf2 helps reduce neuronal loss caused by activated microglia, suggesting that Nrf2 activation is associated with decreased expression of inflammatory cytokines and increased production of anti-inflammatory cytokines, thereby inhibiting neuroinflammation and slowing AD progression [56].

Moreover, mitochondrial function plays a critical role in AD. Nrf2 regulates the expression of anti-apoptotic proteins within mitochondria. When the expression of these proteins is reduced, the activation of the mitochondrial permeability transition pore (MPTP) increases, leading to irreversible mitochondrial dysfunction and damage [57]. Inducing Nrf2 can decrease MPTP activation and maintain mitochondrial function, thereby mitigating age-related mitochondrial dysfunction. Activating Nrf2 through agonists can promote mitochondrial integrity by inducing mitophagy or conferring resistance to oxidative stress-mediated opening of the MPTP [58]. Additionally, Nrf2 interacts with PGC-1α and regulates mitophagy through both p62-dependent and PINK1/Parkin-independent mechanisms, thereby sustaining mitochondrial function [58].

Importantly, numerous studies in AD have focused on targeting ferroptosis by enhancing the antioxidant capacity of Nrf2 and its downstream targets. For example, active compounds extracted from traditional Chinese medicine—such as Rhodiola glycosides [59], artemisinin [60], berberine [61], and forsythiaside A [62]—have been used to target ferroptosis in AD brains. Additionally, ketogenic diets have been reported to improve neuronal ferroptosis in AD mouse models [63]. Various polyphenolic compounds, including chlorogenic acid [64], emodin [64], and eriodictyol [65], have also been widely demonstrated to exert protective effects by activating Nrf2, thereby alleviating ferroptosis. This protection is primarily attributed to the regulation of Nrf2 downstream targets closely associated with ferroptosis, such as SLC7A11, GPX4, and NQO1.

In summary, the antioxidant capacity and ferroptosis processes mediated by Nrf2 and its targets in AD brains are intricately regulated in relation to the pathological features and inducing factors of AD. Practical investigations based on these theoretical frameworks further substantiate the evidence for targeting ferroptosis through the Nrf2 pathway to maintain neuronal viability in AD.

## 4. Exercise and Nrf2

## 4.1 Exercise-Mediated Nrf2 Activation Against Chronic Diseases

Researchers have increasingly focused on early intervention and prevention strategies for AD, aiming to delay the onset and progression of the disease through various preventive approaches. These include, but are not limited to, dietary management (e.g., Mediterranean diet, DASH diet, and consumption of polyphenol-rich foods), psychological and mental interventions (e.g., depression relief, anxiety reduction, and social life enrichment). Among these non-pharmacological strategies, physical activity has garnered significant attention due to its multi-layered health benefits and ease of implementation. Large prospective studies have shown that moderate-to-high intensity physical activity is significantly associated with a reduced risk of cognitive decline [66]. Compared to sedentary lifestyles, individuals who engage in regular exercise have a 20-40% lower probability of developing AD. Clinical trials have further confirmed that moderate aerobic training (e.g., walking, swimming, cycling) not only improves cognitive function in patients with mild cognitive impairment (MCI) but also slows down memory loss and decline in daily living abilities in early-stage AD patients [67].

Regular, scientifically structured exercise is widely recognized for enhancing the body's antioxidant capacity through multiple mechanisms. Maintaining redox homeostasis and resisting oxidative stress have been longstanding focal points in the discussion of "exercise interventions for chronic diseases." Among these mechanisms, the role of Nrf2 remains particularly prominent. This prominence is largely due to the extensive biological evidence demonstrating that exercise-induced activation of Nrf2 regulates numerous AREs, thereby controlling a wide array of antioxidant target genes [63].

AREs are cis-regulatory elements or enhancer sequences located in the promoter regions of genes that encode key antioxidants and various other cell-protective proteins. Notably, the foundational discovery in this field dates back over four decades with the exploration of redox biology in skeletal muscle [68]. Subsequent studies reported significant increases in the abundance of several antioxidant enzymes—such as SOD1, SOD2, catalase (CAT), and GPX—in skeletal muscles subjected to long-term endurance training [69]. These findings have been consistently validated in later research.

While some studies suggest that exercise-induced ROS might counteract antioxidant capacity, this perspective often overlooks critical factors like exercise intensity, duration, and frequency. Long-term, regular, and scientifically tailored exercise—aligned with the body's physiological responses—provenly enhances antioxidant defenses [70]. Consequently, numerous intervention studies targeting chronic diseases have highlighted the benefits of exercise in regulating Nrf2, thereby boosting antioxidant capacity.

In metabolic disorders such as insulin resistance, type 2 diabetes [71], non-alcoholic fatty liver disease [72], and obesity [73], disruptions in redox balance are commonly observed. Consensus among experts and clinical guidelines consistently list exercise as a key non-pharmacological intervention. The underlying mechanism is attributed to exercise-mediated regulation of Nrf2 and the consequent enhancement of antioxidant defenses. These conclusions are supported by a wide range of studies conducted in human and animal models, as well as both in vivo and in vitro experiments.

Similarly, in cardiovascular diseases [74], kidney diseases [75], and various cancers [76], exercise has been reported to modulate Nrf2 activity, yielding protective antioxidant effects. Comprehensive reviews in this area have meticulously documented these findings, further reinforcing the critical role of Nrf2 in exercise-induced antioxidant mechanisms.

In summary, substantial evidence underscores that exercise ameliorates oxidative stress and enhances antioxidant capacity primarily through the regulation of Nrf2. This regulatory role of Nrf2 is integral to the protective effects of exercise against chronic diseases, including Alzheimer's Disease, by maintaining redox homeostasis and mitigating oxidative damage.

## 4.2 Exercise-Mediated Nrf2 Activation Against Cognitive Impairment and AD

Oxidative stress is one of the key factors in the pathogenesis of AD [77]; excessive free radicals and ROS cause neuronal damage, impair normal brain cell function, and exacerbate neurodegenerative changes. Exercise activates the body’s antioxidant system, enhancing cellular antioxidant capacity to help clear these harmful molecules and reduce oxidative stress-induced damage to neurons. Studies have shown that long-term aerobic exercise significantly increases the levels of these antioxidant enzymes, enhancing the cell's ability to clear free radicals. For example, a study found that older adults who participated in regular exercise had significantly higher SOD, GPX, and CAT activity compared to sedentary individuals, highlighting the significant role of exercise in boosting antioxidant defense systems. Moreover, exercise also helps reduce the accumulation of oxidative compounds such as hydrogen peroxide, further alleviating cellular oxidative damage [78]. The neuroprotective effects of exercise are not limited to antioxidant activity. Exercise also improves neuronal function and promotes the survival of nerve cells, combating AD.

Recent studies have begun investigating the mechanisms by which exercise targeting Nrf2 can improve cognitive impairment in AD. MCI, considered a precursor to AD, has been shown to benefit from exercise that activates the AMPK/Nrf2 axis. This activation improves cognitive decline induced by aluminum trichloride and galactose, correlating with enhanced antioxidant capacity in the brain [79]. In the same animal models, exercise-induced activation of Nrf2 inhibits the expression of GSK-3β, reduces mitochondrial damage, and lowers oxidative stress markers in the hippocampus, thereby ameliorating cognitive deficits [80]. As previously discussed, neuroinflammation is a driving factor in AD. In mice with LPS-induced neuroinflammation and cognitive impairment, four weeks of treadmill running upregulated Nrf2 expression and its downstream antioxidant factor HO-1. This upregulation alleviated neuroinflammation and cognitive learning impairments and, importantly, reduced the expression of BACE1. These results were further validated in in vitro experiments [81].

Additionally, metabolic disorders such as diabetes and insulin resistance are recognized as significant risk factors for AD. In elderly diabetic rats induced by a high-fat diet (HFD) and streptozotocin (STZ), eight weeks of voluntary wheel running significantly inhibited oxidative stress in the hippocampus and improved spatial memory [82]. Similarly, another study using HFD-induced endoplasmic reticulum (ER) stress in rat hippocampus found that eight weeks of treadmill running improved cognitive impairment and alleviated insulin signaling disruptions [82]. These findings highlight the ability of exercise to enhance cognitive function by activating Nrf2 signaling, particularly in disease models with various AD-inducing factors.

Furthermore, a population-based study examining sedentary versus active individuals found that those who regularly engage in physical activities have significantly higher Nrf2 expression levels in their red blood cells. These elevated Nrf2 levels negatively correlate with Aβ and tau protein levels [83]. Although these studies did not directly use AD models or samples from AD patients, the association between metabolic abnormalities, neuroinflammation, aging, and AD underscores the importance of Nrf2 in these processes [84].

Importantly, direct AD models have also revealed potential links between exercise and Nrf2. In STZ-induced rat AD models, swimming exercise significantly activated Nrf2 and its downstream antioxidant proteins, thereby inhibiting Aβ deposition and abnormal tau phosphorylation, which led to improved cognitive impairment [85]. Another study using a double transgenic AD model reported that twelve weeks of long-term treadmill running was more beneficial than six weeks. This extended exercise regimen reduced oxidative stress, improved cognitive function in APP/PS1 mice, and attenuated AD-related pathological features, closely associated with Nrf2 activation.

In summary, substantial evidence demonstrates that exercise confers benefits in CNS diseases, especially neurodegenerative diseases, by enhancing antioxidant capacity through the regulation of Nrf2 signaling. This relationship is supported by extensive direct and indirect evidence linking Nrf2-mediated antioxidant improvements to the mitigation of oxidative stress and ferroptosis in AD [86].

In conclusion, the antioxidant capabilities provided by Nrf2 and its downstream targets play a crucial role in inhibiting ferroptosis, a key event driven by oxidative stress. Multiple studies have confirmed that abnormal changes in Nrf2 within AD brains interact with ferroptosis, thereby promoting the progression of AD. Importantly, these findings support the reevaluation of exercise as a powerful intervention to improve oxidative stress in AD brains, thereby rescuing neurons from death, particularly ferroptosis. This provides a robust framework for comprehensively understanding how exercise can alleviate AD.

**Table 1:** Evidence that exercise improves iron metabolism in the CNS and other systems.

Authors	Exercise plan	Disease	Location	Mechanism	Outcomes
***Central Nervous System***					
**Wang et al. [87]**	Exercise-induced irisin	Sepsis-associated encephalopathy (SAE)	Hippocampus	Irisin→Nrf2/GPX4↑	Improvement in cognitive functioning Improvement in neurological function Inhibition of ferroptosis
**Liu et al.[88]**	14 days treadmill training	cerebral ischemia/reperfusion (I/R) injury	cerebral cortex	Nrf2↑→SLC7A11↑→GPX4	Neurological Impairment Recovery Cortical lipid peroxidation mitigation Inhibition of ferroptosis
**Chen et al. [89]**	4 weeks (20 days) treadmill training	Traumatic brain injury	cerebral cortex	GPX4↑STING↑	Improvement in cognitive functioning Inhibition of ferroptosis
**Fang et al. [90]**	4 weeks (20 days) treadmill training	Depressive	Hippocampus	miR-150-5p -Xc-/GPx4	Decreased depression-like behavior Inhibition of ferroptosis
***Aging-related disease***					
**Wang et al. [91]**	10 months of treadmill training then stop training	Aging	Aging skeletal muscle	Keap1↑, Nrf2↓, SLC7A11↓, GPX4↓	Skeletal muscle atrophy oxidative stress Increased inflammation Increased ferroptosis
**Yuan et al. [92]**	18 months treadmill training	Aging	Gallbladder	p53↓, p21↓, Nrf2↑, SLC7A11↑, GPX4↑	Decreased inflammatory factors Decreased apoptosis oxidative stress Inhibition of ferroptosis
**Tao et al. [93]**	4 weeks (28 days) treadmill training	Postmenopausal osteoporosis	Serum and bone	Irisin/Cav1↑ AMPKα↑,AMPK↑,Nrf2↑, HMOX1↑, Fpn↑	Increased osteoblast proliferation Inhibition of ferroptosis
**Han et al. [94]**	4 weeks (20 days) treadmill training	Osteoarthritis	Knee	NF-κB↓, p65↓, p53↓, Nrf2↑, SLC7A11↑, GPX4↑	Reduces cartilage matrix degradation Inhibition of ferroptosis

## 4.3 Exercise-Mediated Nrf2 Activation Against Ferroptosis

As discussed above, oxidative stress resulting from abnormal iron metabolism plays a crucial role in driving the progression of AD through ferroptosis in the brain. Regular and structured physical activity, known for its antioxidant effects, is highly likely to be involved in the ferroptosis process and iron metabolism in the AD brain. More importantly, this process is mediated by the Nrf2 signaling pathway.

As shown in Table 1. Previous studies have provided partial evidence, as summarized in the table below, which outlines the current understanding of how exercise can resist ferroptosis in the central nervous system and other systems during aging. However, it is important to note that there is currently no complete body of evidence that definitively demonstrates the impact of exercise on ferroptosis in the AD brain. Most studies so far focus on investigating the antioxidant capacity in the AD brain, rather than directly linking exercise to ferroptosis.

**Figure 3.**
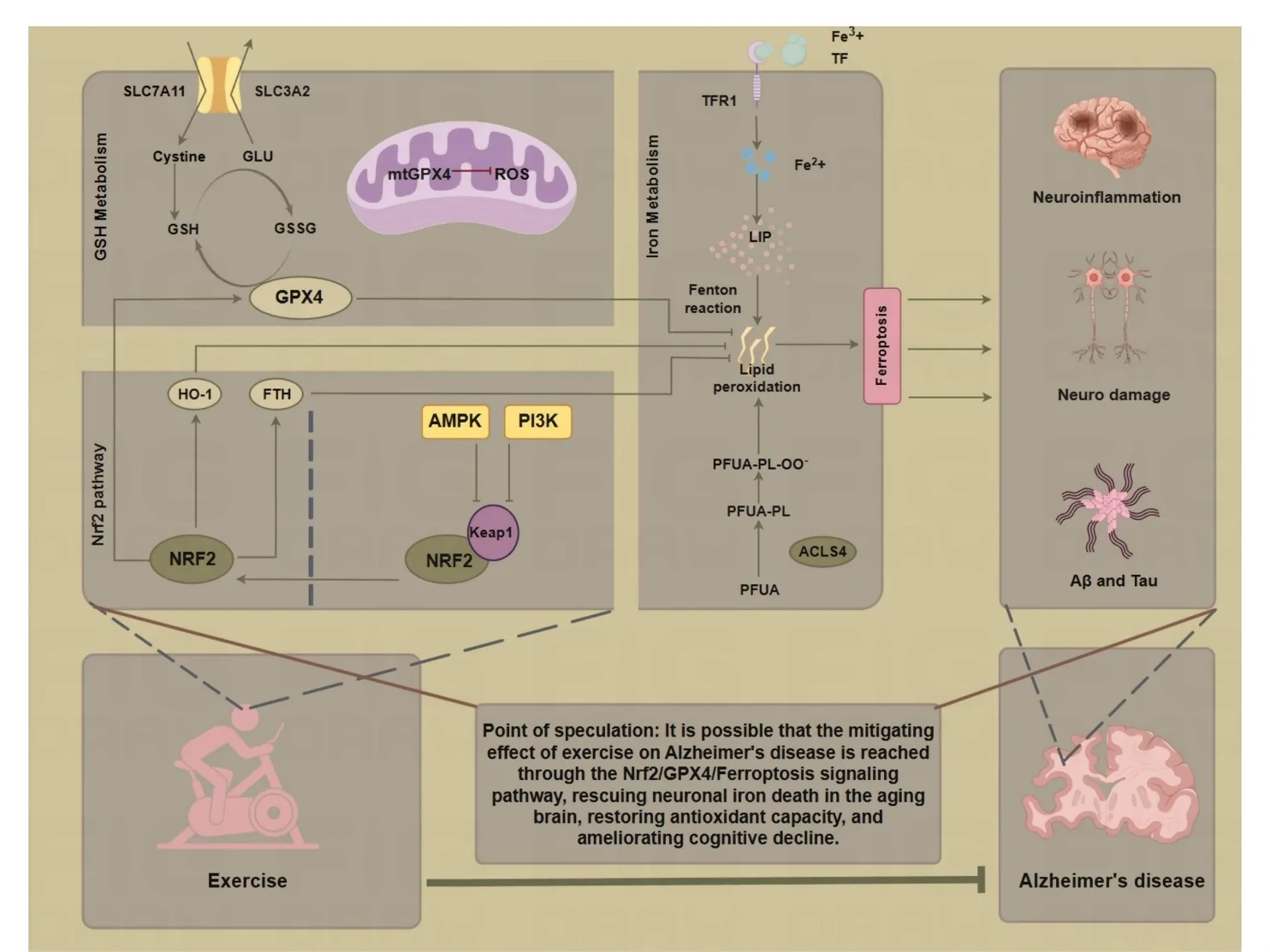
**Possible Mechanisms of Exercise Ameliorating Ferroptosis in AD Brain**. Point of speculation: It is possible that the mitigating effect of exercise on Alzheimer's disease is reached through the Nrf2/GPX4/Ferroptosis signaling pathway, rescuing neuronal iron death in the aging brain, restoring antioxidant capacity, and ameliorating cognitive decline. Under the influence of exercise, AMPK is activated and promotes the phosphorylation of Keap1, leading to the dissociation of Nrf2 from Keap1 and facilitating its nuclear translocation. Additionally, exercise upregulates the PI3K-Akt signaling pathway, which inhibits GSK-3β activity, thereby reducing Nrf2 phosphorylation and degradation.

In summary, as shown in Figure 3, the antioxidant capacity conferred by Nrf2 and its downstream targets plays a unique and crucial role in inhibiting oxidative stress, which is a key event in ferroptosis. Multiple studies have confirmed that abnormal alterations in Nrf2 within AD brains interact with ferroptosis, thereby promoting the progression of AD. Most importantly, these findings provide a robust foundation for re-examining the role of exercise in mitigating oxidative stress within AD brains. By improving oxidative stress, exercise can effectively rescue neurons from death, particularly ferroptosis, thereby offering a comprehensive explanation for the beneficial effects of exercise in alleviating AD.

## 5. New Perspective: Exercise Alleviates Ferroptosis in Alzheimer's Disease through Nrf2 Signaling Modulation

Although the above content has thoroughly demonstrated the relationship between exercise, AD, and ferroptosis, the existing evidence mainly focuses on two separate topics: the relationship between exercise and AD, the relationship between ferroptosis and AD, and the relationship between exercise and ferroptosis. Therefore, further research is needed to elucidate the interconnections among the three, particularly focusing on Nrf2, a key signaling pathway that links them all.

**Figure 4.**
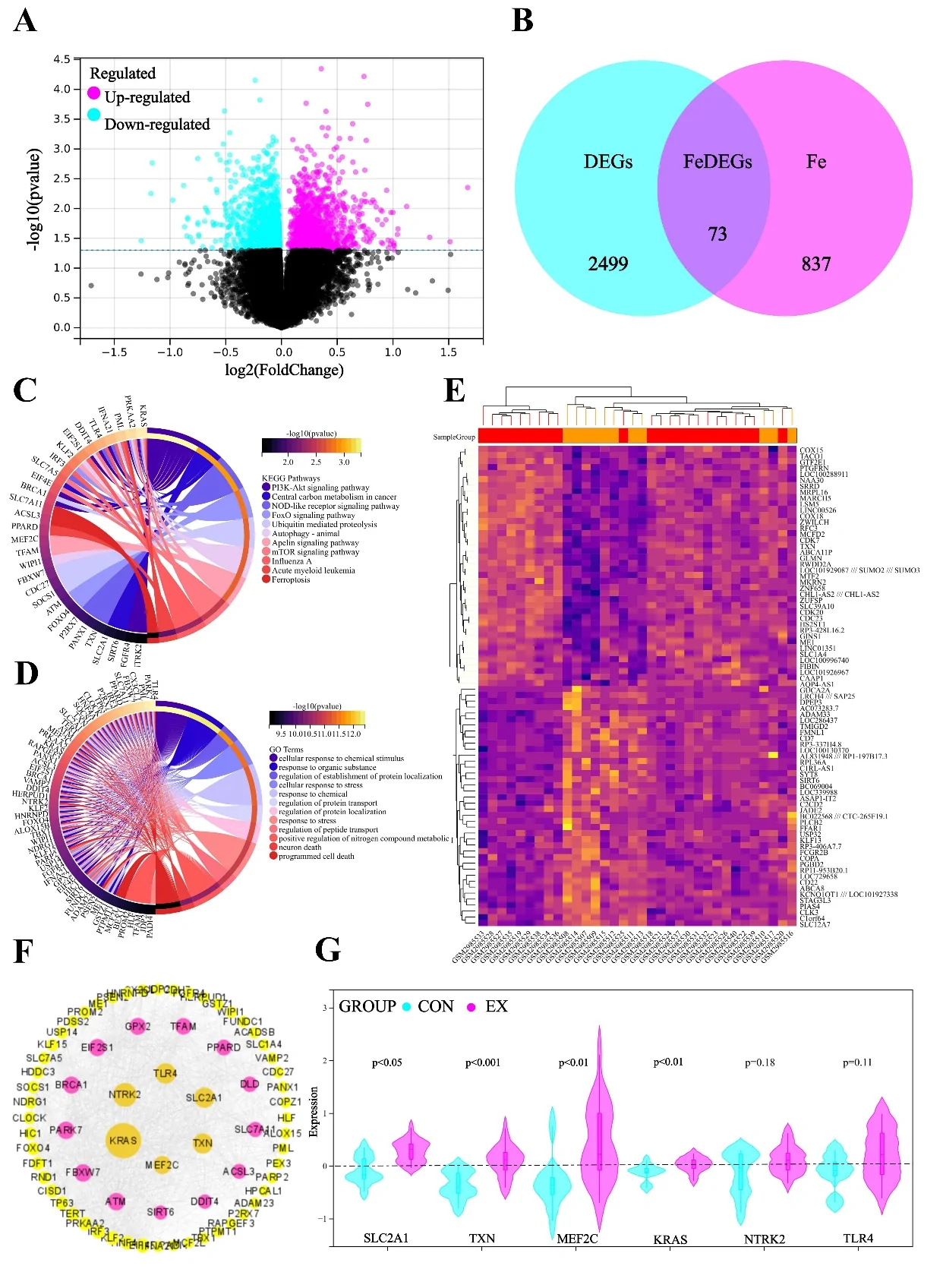
**Identification of key genes associated with ferroptosis in athletic/non-athletic AD individuals using bioinformatics**. (**A**) Differential gene volcano map, (B) Intersecting genes of differential genes with iron death genes, (C) KEGG analysis, (D) GO analysis, (E) Heat map of differential genes, (F) PPI protein interaction network, G. Relative expression of Hub genes

In light of this, we have conducted preliminary validation efforts to provide additional evidence in this area. Using the GSE110298 expression profile found in the GEO dataset, which consists of hippocampal tissue collected 24 hours post-mortem from subjects, we applied the limma method (log_2_FC = 1, P < 0.05) to compare the experimental group (EX group) to the control group (CON group). The results showed 1,142 upregulated genes and 1,430 downregulated genes in the EX group, as shown in Figure 4A, E.

We obtained ferroptosis-related genes (Fe) from an online database, which collects genes currently identified in the literature as being closely related to ferroptosis, including driver genes, marker genes, and inhibitor genes. The differentially expressed genes (DEGs) were intersected to identify 73 ferroptosis-related DEGs (FeDEGs), as shown in Figure 4B. These FeDEGs were further subjected to pathway enrichment analysis using KEGG and GO. The results are illustrated in the figures below.

In the KEGG pathway analysis, we found that the FeDEGs were enriched in the PI3K-Akt signaling pathway, NOD signaling pathway, FoxO signaling pathway, mTOR signaling pathway, and PPAR signaling pathway, as shown in Figure XC. The biological process (BP) enrichment analysis highlighted processes such as regulation of protein transport, protein localization, programmed cell death, cellular response to stress, and regulation of cellular metabolic processes. In the cellular component (CC) enrichment analysis, we observed enrichment in cellular locations such as cytoplasmic ribonucleoprotein granules, chromosomes, mitochondria, and the whole membrane. The molecular function (MF) enrichment analysis revealed processes including transmembrane transport of sulfur amino acids, oxidoreductase activity, lipid binding, transcription factor binding, and protein dimerization activity, as shown in Figure 4D.

We performed a protein-protein interaction (PPI) analysis on the 73 FeDEGs using the STRING online tool, and the results were imported into Cytoscape software to identify HUB genes. The analysis revealed that KRAS, NTRK2, TLR4, SLC2A1, TXN, and MEF2C are considered HUB genes in the PPI network of FeDEGs, as shown in Figure 4F. A box plot was then generated to observe whether there were differences in the expression of these HUB genes between the groups, using non-parametric tests. SLC2A1, TXN, MEF2C, and KRAS were found to be highly expressed in the EX group, with statistically significant differences between the groups, as shown in Figure 4G.

Glucose Transporter 1 and Its Role in Antioxidant Defense via Glucose Uptake: Glucose transporter 1 (SLC2A1), by promoting glucose uptake, indirectly supports the cell’s antioxidant defense mechanisms [95]. AMPK is a key cellular energy sensor, and when cellular energy levels decrease, AMPK is activated to enhance glucose metabolism. As a glucose transporter, the function of SLC2A1 may be regulated by AMPK, especially in situations involving exercise or significant energy expenditure [96]. Activation of AMPK may increase the expression of SLC2A1, thereby enhancing glucose uptake and providing more energy for the cell. This supports antioxidant responses and reduces the occurrence of ferroptosis.

Thioredoxin (TXN) as an Antioxidant Factor in Cellular Protection: Thioredoxin (TXN) functions as an antioxidant factor by scavenging ROS within the cell, alleviating oxidative stress, and protecting cells from oxidative damage [97]. Nrf2 is an upstream regulator of TXN. Nrf2 is activated when cells experience oxidative stress, promoting the expression of antioxidant genes, including TXN [98]. Nrf2 is not only closely associated with ferroptosis but also protects cells from oxidative damage by enhancing antioxidant responses during exercise [99]. Therefore, the interaction between TXN and Nrf2 may play an important role in the process of ferroptosis.

Muscle Enhancer Factor 2 and Its Role in Antioxidant Defense: Muscle enhancer factor 2 (MEF2) is involved in regulating neuronal differentiation and survival. By activating antioxidant genes, MEF2 helps cells cope with oxidative stress [100]. AMPK, by activating MEF2, may enhance the cell’s antioxidant capacity. AMPK activation helps cells respond to oxidative stress during low-energy states, and MEF2 activity provides neuroprotective effects, potentially reducing neuronal death, particularly in neurodegenerative diseases [101]. MEF2 may also indirectly regulate iron metabolism, thereby mitigating the risk of ferroptosis.

A Network of Interconnected Factors Involved in Oxidative Stress Response: SLC2A1, TXN, MEF2, AMPK, and Nrf2 form an interconnected network in the cell’s response to oxidative stress, energy metabolism, and antioxidant defense. When cells experience oxidative stress or energy shortage, AMPK is activated and promotes glucose uptake by upregulating SLC2A1, providing the cell with more energy. Simultaneously, AMPK activates MEF2 to enhance antioxidant defenses. TXN, as a downstream target of AMPK and Nrf2, further strengthens the cell’s antioxidant capacity, reducing ROS accumulation. Nrf2 provides additional protection by activating a series of antioxidant genes, including TXN, thereby mitigating oxidative damage. Through this mechanism, these five genes not only coordinate cellular energy metabolism and antioxidant responses but may also jointly regulate iron metabolism, inhibiting ferroptosis.

## 6. Critical Analysis and Future Research Directions

In this study, it is essential to critically analyze the dataset used, particularly focusing on data quality and its relevance to the study hypotheses. Firstly, the original study was logically and scientifically designed, providing a solid foundation for our hypothesis.

Although our hypothesis suggests that regular, scientifically structured exercise can inhibit ferroptosis in the brains of AD patients, the original study did not clearly define the scope of high-intensity exercise. Therefore, we cannot immediately conclude the scientific validity of exercise intensity within our hypothesis. This highlights the need for further experimental research, especially regarding exercise intensity, duration, and frequency, as high-intensity exercise may potentially induce ferroptosis [102, 103].

Secondly, our hypothesis is based on transcriptome sequencing of hippocampal tissue. However, studies indicate that the four cell types in the CNS—microglia, oligodendrocytes, astrocytes, and neurons—respond differently to ferroptosis. Whole-tissue transcriptome sequencing may obscure these differences, thus single-cell sequencing analysis is necessary to distinguish the responses of different cell types.

Furthermore, additional research is required, particularly expanding transcriptome sequencing to explore variations across different regions and ethnic groups.

Addressing the above issues, future studies should conduct relevant experiments to validate our hypothesis. Specifically, multiple avenues need to be explored to uncover how exercise regulates Nrf2 to improve ferroptosis in AD brains, thereby alleviating AD. Firstly, animal model studies are urgently needed to validate the targets identified through bioinformatics analysis that are closely related to oxidative stress and ferroptosis, using techniques such as Western blot and PCR for initial verification at the gene expression and protein levels. Subsequently, more complex experiments should be designed to explore the complete pathways, utilizing various AD models like APP/PS1, 3×Tg, or 5×FAD, and employing qualitative or quantitative methods to analyze our proposed hypotheses. Importantly, reverse validations are necessary to clarify the complex in vivo signaling networks, using antagonists or agonists, siRNA, and lentiviral transfection to validate specific targets.

Moreover, although animal models strive to mimic the pathogenesis of human AD, there are still differences. Therefore, researchers with sufficient resources may explore human AD samples, such as cerebrospinal fluid or autopsy samples, despite the difficulty in obtaining them. Alternatively, in the design of large-scale cross-sectional or cohort studies, biochemical markers in the blood that reflect iron homeostasis, such as transferrin, transferrin receptor, transferrin binding capacity, or transferrin saturation, could be considered to evaluate our hypothesis [104, 105]. Notably, other metal ions like copper and zinc, although essential like iron, may also play a role in the discussion of exercise preventing AD. Similar to iron, exceeding homeostasis levels of these metal ions can trigger adverse events, such as cuproptosis and zinptosis [106-109].

In summary, despite some points requiring special attention, our study's hypothesis provides a direction for advancing and clarifying how exercise regulates Nrf2 to improve ferroptosis in AD brains, thereby alleviating AD. Moving forward, more studies based on this hypothesis are needed to enrich the research conclusions.

## 7. Conclusion

Based on the above content, we believe that current research on exercise for the prevention and alleviation of AD lacks a focus on how exercise may mediate prevention and relief by regulating ferroptosis in the AD brain, particularly through the Nrf2 signaling pathway. We hope that future research will scientifically and rationally verify the hypotheses proposed above, to provide stronger evidence for AD prevention and alleviation, ultimately offering effective guidance for clinical interventions in AD.

## Data Availability

The data in this study were drawn from the Database for Gene Expression Omnibus (GEO) program (Home - GEO - NCBI).
